# Perspectives on substance abuse among nursing students and educators in Eastern Cape, South Africa

**DOI:** 10.4102/hsag.v31i0.3302

**Published:** 2026-09-28

**Authors:** Nomthandazo M. Sululu, Allison H. Du Plessis

**Affiliations:** 1 Department of Nursing Science, Faculty of Health Sciences, Nelson Mandela University, Gqeberha, South Africa

**Keywords:** perspectives, nurse educators, nursing students, substance abuse, nursing education institutions

## Abstract

**Background:**

Substance abuse in nursing raises ethical and legal concerns, with 10% – 20% of students affected. Research indicates these behaviours often continue into professional practice and need to be addressed.

**Aim:**

To explore and describe the perceptions of nurse educators and student nurses regarding substance abuse and to make recommendations to address substance abuse at a public nursing college in the Eastern Cape.

**Setting:**

A public nursing college education institution, comprising five main campuses and 18 sub-campuses in the Eastern Cape.

**Methods:**

A qualitative, exploratory and descriptive design was used. Purposive sampling was used to select participants, and data were collected through three nurse educator focus groups and three student nurse focus groups. Nurse educator focus groups consisted of four participants each, and student focus groups consisted of six participants each. The data were analysed using thematic analysis. Trustworthiness was established using Guba and Lincoln’s criteria.

**Results:**

Three themes emerged: (1) Nurse educators and nursing students perceive substance abuse as a complex issue influenced by diverse personal and environmental factors, (2) Substance abuse impacts student nurses across three domains of their well-being and (3) The management of substance abuse within the education institution lacks comprehensive, structured and consistent strategies.

**Conclusion:**

Substance use among nursing students is fundamentally a health and well-being issue that requires professional and structured interventions.

**Contribution:**

Describing the perceptions of nurse educators and nursing students led to a set of recommendations to address substance abuse among student nurses.

## Introduction

A high prevalence of burnout among nursing students is linked to demanding academic and clinical workloads (Kong et al. 2023). Substance misuse within the nursing profession raises ethical and legal concerns. Nurses are expected to model healthy behaviours that promote positive patient outcomes and uphold professional ethics, thereby fostering trust among patients, families and the wider community. Research indicates that substance misuse persists beyond graduation, with professionals continuing such behaviours in their practice (Bahji et al. 2022), leading to legal consequences, including misuse of prescription drugs and patient neglect. Although the college under study’s governance structures required nurse educators to identify and report concerning student behaviours in both academic and clinical settings, a significant gap existed in the institution’s ability to effectively support nursing students struggling with substance abuse. Despite growing concerns regarding substance abuse among nursing students, little is known about how nurse educators and nursing students perceive substance abuse and its management within South African nursing education institutions, particularly in the Eastern Cape province. While students could approach college management for general matters, no specialised framework was available to address the complex and multifaceted needs of this vulnerable group. This shortcoming was emphasised when a pattern of drug and alcohol abuse emerged among nursing students, evidenced by declining academic results, complaints from clinical facilities and investigations into adverse incidents involving students. The impact of this gap became an urgent ethical issue when students were expelled without receiving proper treatment or follow-up care, leaving their physical, psychological, social, economic and spiritual well-being unaddressed. The National Department of Health’s strategic plan for substance abuse called for strengthening, monitoring, evaluating and researching substance use disorder (Health Sector Drug Master Plan 2019–2025). This strategic plan specifically called for improvement of institutional capacity for prevention, early detection, treatment, rehabilitation and after-care services for substance use disorders as one of the major goals. Although the college staff were expected to identify and refer students showing unexplained behaviours, there was no institutional support system in place to receive and appropriately assist those students once identified. This gap between the duty to recognise the problem and the lack of targeted intervention mechanisms revealed a serious flaw in the college’s student support system, especially for those most in need of early and specialised care. This study aimed to establish the perceptions of nurse educators and student nurses regarding substance abuse. The research objectives were: (1) to explore and describe the perceptions of nurse educators, (2) to explore and describe student nurses’ perceptions concerning substance misuse and its management at a public nursing education institution in the Eastern Cape. Recommendations were made based on the study’s findings and (3) to make recommendations to address substance abuse at the public nursing college in the Eastern Cape. The study also highlighted the importance of personal development by providing nursing students with effective coping strategies to manage academic and emotional stress, thus creating a supportive learning environment.

### Literature review

Substance abuse represents a significant social issue among students in educational institutions worldwide. According to the National Survey on Drug Use and Health (NSDUH) (Substance Abuse and Mental Health Services Administration [SAMHSA] 2023), approximately 49% of students aged 18 to 22 years in educational settings reported consuming alcohol. About 29% of these students engaged in binge drinking during the same period. The NSDUH notes that cannabis misuse remains prevalent, with 39.5% of college and university students using marijuana in 2023. Furthermore, 22.2% of students have used an illicit drug within the past month (Substance Abuse and Mental Health Services Administration [SAMHSA] 2023). Similarly, Vorster et al. (2019) observed increasing alcohol consumption among second- and third-year medical students in South Africa (SA). The most used substances by SA students include alcohol (80.6%), cannabis (46%) and ecstasy (5.3%) (Netshiswinzhe et al. 2023). The United Nations Office on Drugs and Crime (2025a, 2025b) reported in their World Drug Report that approximately 244 million people, or 4.6% of individuals aged 15 to 64 years, used cannabis in 2023. Although specific data on substance abuse among nursing students in SA are limited, institutional observations indicate a significant prevalence within this public nursing education institution. An informal survey conducted during a coronavirus disease 2019 (COVID-19) wellness week in 2021 among nursing students showed that between 10% and 20% of them experience substance addiction. This pattern aligns with national trends of alcohol and drug misuse among individuals aged 15 to 25 years, who constitute 33% of the drug-using population (Peltzer & Phaswana-Mafuya 2018). Nursing students have attributed their substance use to academic stressors, mental health pressures and ineffective or unenforced institutional policies (Rattner 2021). While nursing students are aware of the risks associated with substance abuse, early recognition of warning signs is crucial. Behavioural indicators such as absenteeism, tardiness and withdrawal from peers and faculty can signal a need for intervention (Kong et al. 2023). Furthermore, Hirani et al. (2022) identified the learning environment, faculty attitudes and family dynamics as influential factors in substance use among nursing students. Therefore, as future healthcare providers, it is essential to prioritise mental well-being throughout training. To address these challenges, Netshiswinzhe et al. (2023) proposed a comprehensive model integrated into Alternative to Discipline (ATD) programmes. The model emphasises psychosocial support and collaboration among law enforcement, communities and families to reduce substance abuse. Importantly, the roles of lecturers and professional nurses at clinical sites are highlighted, particularly in fostering supportive relationships, creating conducive teaching and learning environments. Recently, the Western Cape High Court’s ruling on cannabis prohibition revisited debates over individual rights versus societal consequences (Republic of South Africa 2020). While the legalisation of private cannabis use has been upheld in South Africa, and the *Liquor Act 59 of 2003* regulates the manufacture, distribution and sale of liquor and includes provisions to prevent underage drinking, and the National Liquor Policy (Department of Trade and Industry 2016) emphasises reducing alcohol-related harm among youth by proposing stricter advertising laws, raising the legal drinking age (suggested to 21), the relevance of this legislative landscape to nursing education lies in the gap between policy intent and institutional reality. Nursing students are predominantly young adults within the same age cohort identified as vulnerable to cannabis-related cognitive and academic harm, and the same weak enforcement of liquor advertising restrictions, outlet density limits and age verification that allows alcohol to remain ‘readily available and permissive’ for the general youth population applies equally to students enrolled at nursing colleges. These legal frameworks are not designed to regulate behaviour within educational institutions, and thus, nursing colleges cannot rely on broader legal frameworks to protect students from substance use or to compel early identification and intervention. This places the responsibility for prevention, detection and support squarely on the institution itself, a responsibility the college in this study was not structurally equipped to fulfil. Substance abuse among nursing students remains a concern in South African academic institutions (Republic of South Africa 2020) because of academic stress. Studies on the impact of substance abuse among adolescents have highlighted adverse behavioural outcomes, including academic underperformance, conflicts with authority figures and engagement in risky behaviours (Mokwena & Setshego 2021). Similarly, anecdotal evidence from the public nursing education institution where the research was conducted suggests that nursing students who abuse substances face conflicts with authority figures and engage in risky behaviours. Furthermore, there is a correlation between substance use and poor attendance, which, in turn, affects academic performance and progression (Brito et al. 2024; Duckworth et al. 2023; Rodríguez-Sáez et al. 2025). In the absence of protocols to manage substance abuse at this public nursing education institution, counselling and referrals by nurse educators have had an insignificant effect on the behaviour of substance-abusing nursing students. This study of the perspectives of nurse educators and nursing students on substance abuse at a nursing education institution in the Eastern Cape offers social value by generating scientific knowledge that can inform recommendations for nursing education institutions and guide targeted interventions to address substance abuse. Such interventions may further enhance ethical awareness and promote responsible professional behaviour among nursing students, qualities they can carry into their careers. The findings emphasise the need to establish peer support and referral systems to enable early identification and assistance for nursing students struggling with substance abuse. This approach may help prevent academic and clinical misconduct.

### Research setting

The study was conducted at a public nursing education institution in the Eastern Cape province, which comprised five main campuses and 18 sub-campuses. This institution was chosen because the college governance structures called on nurse educators to identify and report on student matters that affect graduate outputs, which was, at that time, a concern. On different occasions, student nurses approached the nurse educators for help. This public nursing college promotes the development of students from disadvantaged backgrounds and therefore has a diverse student population. At the time of the research, the institution offered various nursing education programmes, including the 4-year comprehensive diploma programme (R425), before it was accredited as a higher education institution. All students studied full time, with many residing in unsupervised nurses’ homes on campus sites. The student population often lacked social support systems and was funded by the government.

## Research methods and design

### Design

A qualitative approach with an exploratory, descriptive and contextual design was employed for this study to explore and describe how nurse educators and nursing students perceive substance abuse and its management within South African nursing education institutions. A qualitative approach was appropriate, as the topic was sensitive and required real voices to capture the deep social nuances that questionnaires cannot. Focus groups were ideal for gaining a rich understanding from the diverse general nursing student and nursing educator population. Individual interviews would not have been ideal because they would put individuals at risk of sharing sensitive personal information, creating ethical dilemmas for the researcher in reporting the findings. The researcher anticipated that individual interviewees would be more likely to withdraw from the study after data collection, having shared personal and sensitive information.

### Theoretical framework

The researcher used the Social Stress Model of Substance Abuse by Rhodes and Jason (1989) to understand nursing students’ perceptions of substance abuse. This model differs from other theoretical approaches by considering wider social influences on adolescent behaviour. It suggests that adolescents turn to substance use as a coping mechanism in response to stressors such as academic pressure, clinical workload, financial difficulties and exposure to illness and trauma during training. This supports the model’s claim that substance abuse is a reaction to overwhelming stress. The model indicates that the risk of substance abuse decreases when individuals have sufficient social skills to resist substance misuse and access to community resources, positive role models and opportunities (Rhodes & Jason 1990). Interventions reflecting the model’s emphasis on strengthening protective mechanisms could inform recommendations for nursing education institutions and highlight the need for a structured, professional response to substance abuse. By applying the model, the study examined how stress, social dynamics and institutional support (or lack thereof) interact to influence substance abuse among nursing students, providing a strong theoretical basis for recommending comprehensive, empathetic interventions that address both the causes and consequences of substance abuse in nursing education.

### Population and sample

This public nursing college, the focus of the study, has a diverse student and faculty population. At the time of data collection, the college served a total student nurse population of 1773 and 68 nurse educators. The entire student and educator populations were the target. Purposive sampling was used to select participants from two groups: (1) nurse educators and (2) nursing students. Tenny, Brannan and Brannan (2022) highlight that purposive sampling is best adopted when studying stigmatised groups or populations where findings are likely to be difficult to obtain by ordinary means. Campus heads functioned as gatekeepers, allowing the researcher access to distribute information about the research to the entire population. Potential persons from the population who indicated interest in participating could contact the researcher, after which they received detailed information, including guidelines, confidentiality agreements and consent forms. Upon contact, the researcher assigned numbers to interested participants from each of the three campuses, using a lottery method to reduce selection bias within the purposive pool. Once the interested persons signed the consent, were they considered enrolled as participants. The researcher assigned numbers to all eligible participants from each of the three campuses and used a lottery to select them upon contact. Separate focus groups were held for nurse educators and nursing students to ensure that students could share their perceptions freely without fear of nurse educators. The inclusion and exclusion criteria are presented in Table 1.

**TABLE 1:** Inclusion and exclusion criteria.

Inclusion	Exclusion
**Nurse educators** Nurse educators with at least 2 years of teaching experience in the nursing college. Nurse educators who engage in college committees dealing with students’ academic matters. Nurse educators who provide clinical accompaniment.	Campus heads were excluded because they are not directly involved in teaching students.
**Student nurses** Students over the age of 18 were selected to avoid issues with minors. Students who voluntarily consent to participate to ensure ethical compliance and data validity. Students who are actively registered at the time of data collection to ensure the data reflects current experiences rather than retrospective or outdated contexts. Nursing students at all year levels in the 4-year diploma programme because substance abuse occurs across year levels. Students who voluntarily consent to participate to ensure ethical compliance and data validity. Students who have participated in classroom learning and/or clinical placements.	Students on abeyance were excluded. Students who were in therapy for substance abuse or were known substance abusers were excluded.

### Data collection

Focus groups were selected for their ability to capture nuanced perspectives, generate interactive discussions and validate issues through peer exchange, making them well suited for exploring shared norms and behaviours (Hennink, Hutter & Bailey 2020). Data were collected in June and July 2022 from three main campuses. The researcher arranged interviews via gatekeepers. The study comprised six focus groups: two with nurse educators (one pilot and one main focus group) and four with nursing students (one pilot and three main focus groups). The main study included one focus group of six nurse educators from the three campuses and three groups of nursing students, with four participants (Campus A), five (Campus B) and six (Campus C). As no changes to the interview schedule were needed after the pilot semi-structured interviews, the pilot focus group interview data were also included because it was rich data. A total of six (*n* = 6) focus group interviews were used for data analysis and inclusion into the study. Semi-structured focus group interviews were conducted face to face. Openness and freedom were ensured through a round-table discussion; ground rules were established at the start of each focus group, and it was emphasised that there are no right or wrong answers and that all opinions are equally valid, which contributed valuable information to the study. Each focus group interview lasted 1 h to 1 h and 15 min. Interviews were audio-recorded and transcribed verbatim. Field notes recorded non-verbal cues such as gestures and expressions. An experienced facilitator conducted the interviews, supported by a moderator who managed group dynamics and coordination and encouraged diverse participation. Each focus group started with the main research question indicated in Table 2. Nurse educators were asked about their understanding of substance abuse among nursing students, and nursing students were asked about their understanding of substance abuse in general and within the student population. Table 2 presents the interview probing questions to use if relevant information was not volunteered. Data collection continued until saturation was achieved, and no new themes emerged.

**TABLE 2:** Semi-structured focus group interview questions.

A. Interview questions for nurse educators	B. Interview questions for nursing students
**Main question** What is your understanding of substance abuse among nursing students?	**Main question** What is your understanding of substance abuse in general and within the student population?
**Examples of probing questions that were asked if information was not volunteered.** What signs or behaviours might indicate that a student is struggling with substance use? In your opinion, how does substance abuse affect students’ academic performance and clinical practice? What are the professional and ethical concerns regarding nursing students using substances? What mechanisms are currently in place at the educational institution to address or manage substance abuse? What kind of support do you think students need when facing substance-related issues? What interventions or support systems would you recommend to improve how substance abuse is addressed in nursing education?	**Examples of probing questions that were asked if information was not volunteered.** What do you think are the main reasons why some students may turn to drugs or alcohol? Are there specific stressors in nursing education that might contribute to substance use? How does substance abuse affect you academically, socially or emotionally? If you or a peer were struggling with substance use, how comfortable would you feel seeking help? What barriers might prevent students from accessing help for substance use? What changes do you think the educational institution should make to better support students dealing with substance abuse?

Before the main study, a pilot study was conducted comprising two focus groups: one with six nurse educators and one with six nursing students. The pilot took place at a remote main campus away from the primary study sites to reduce the risk of data contamination. The data collection methods used in the pilot closely matched those planned for the main study. The focus group interviews were held in-person on separate occasions in the boardrooms of the three main campuses. As no modifications were required to the interview guide, procedures or data collection process, and the pilot participants met the study inclusion criteria, the pilot data were included in the final analysis. Consequently, data from six focus groups (two pilot and four main study focus groups) were included in the final analysis.

### Data analysis

Data analysis involved transcribing all collected data verbatim using Otter.ai, as it is well suited to qualitative descriptive studies. The researcher verified the accuracy of the transcripts by listening to the digital recording while following along. Tesch’s method, as referenced in Creswell (2014), was employed to analyse the data. Coding was done manually, and deductive reasoning was used to move from general themes to specific conclusions. Coding was an iterative process where re-reading and re-coding were done to refine the main themes and subthemes. The procedure followed the eight steps proposed by Tesch, also cited in Creswell (2014), which were to:

- Gain an overall understanding by carefully reading all transcriptions and noting initial ideas.
- Analyse each document individually, seeking underlying meanings and jotting down thoughts in the margins.
- After completing this task for multiple participants, compile a list of topics and group similar ones together, organising them into major, unique and residual categories.
- Use this list to revisit the data, condensing topics into codes and assigning them to relevant segments of text. Assess this initial organisational scheme for the potential emergence of new categories.
- Refine topics into categories by finding the most descriptive wording, grouping related topics and establishing interrelationships between categories.
- Finalise category abbreviations, using letters for codes.
- Gather data corresponding to each category and conduct preliminary analysis.
- If needed, reevaluate and recode the existing data.

An independent qualitative researcher coded the data, and consensus discussions were held to resolve discrepancies.

### Trustworthiness

The methods that support the analysis of qualitative studies, namely credibility, confirmability, dependability and transferability, as outlined in Guba and Lincoln’s criteria (cited in Morse 2015), were applied to ensure the trustworthiness and quality of the research data. Credibility was enhanced by using a voice recorder to capture data, observing participants’ actions and mannerisms during group discussions and asking clarity-seeking questions when responses were vague. All data were transcribed verbatim, verified for accuracy by the researcher and analysed systematically. Data were validated by participants (member checking), and peer debriefing was conducted with colleagues to review and critique the research process and interpretations. Confirmability was ensured through field notes, verbatim transcriptions and the involvement of an independent coder with whom consensus meetings were held to agree on the main themes. Dependability was achieved by adhering to qualitative research methods. Rigorous documentation of the research process was kept, creating an audit trail. Research decisions, procedural changes and analytical choices were discussed with research supervisors. The researcher attended faculty-led research sessions to enhance methodological knowledge and maintain a clear audit trail, sought input from the research supervisor and conducted a consensus meeting with the supervisor and an independent coder to verify themes. Transferability was supported by providing detailed descriptions of the research methods and context. The researcher was from a campus that did not participate in the research study to maintain neutrality and prevent position from being an influential factor. Researcher bias was further avoided by keeping a reflexive journal throughout data collection and analysis to track personal reactions and assumptions as they arose. During the pilot semi-structured interviews, an opportunity arose to assess researcher influence. A literature review was conducted after data collection to ensure that prior knowledge during interviews and coding did not override participants’ verbatim expressions. The researcher also ensured the integrity of the research data by submitting the interview recordings as they were collected to the research supervisor, who discussed interview quality throughout the data collection period.

### Ethical considerations

Approval for the study was obtained from two credible research ethics bodies overseeing human research: the Nelson Mandela University Research Ethics Committee for Human (H19-HEA-NUR-007) and the National Health Research Ethics Committee. The research was conducted in accordance with the Ethical Principles and Guidelines for the Protection of Human Subjects of Research, as outlined in the Belmont Report of 1978 (United States. National Commission for the Protection of Human Subjects of Biomedical and Behavioural Research 1978). This important report highlights three core ethical principles that guide research: respect for persons, beneficence and justice. Before participating, focus group members had to sign an informed consent form. Confidentiality cannot be fully guaranteed in group settings; therefore, each participant signed a confidentiality agreement. Although participants within the same focus group knew each other, those in different groups remained anonymous. Each participant was assigned a number for identification during interviews, and the transcripts for each focus group interview were numbered alphabetically to protect anonymity. After the focus group interviews, those who revealed and acknowledged previous drug use behaviour were privately approached and advised to contact the gatekeeper, through whom a referral pathway was established. The digital recordings, verbatim transcripts, scanned consent forms and confidentiality forms were stored electronically and protected with a password. Research data were uploaded to the university’s secure site in accordance with the ethics committee’s requirements. The researcher adhered to ethical principles to ensure authenticity and truthfulness. As a nurse educator familiar with the nursing education institution, the researcher applied reflexivity to maintain neutrality and objectivity.

## Results

Focus group interview findings for student nurses and nurse educators were synthesised by identifying relationships, topics and themes. Three main themes, with subthemes, were identified. The themes from the different focus groups were synthesised into one set of findings. The themes are presented in Table 3. The findings from the focus group interviews are reported and participant demographics are presented in Table 4 and Table 5.

**TABLE 3:** Themes and subthemes.

Themes	Subthemes
1. Nurse educators and nursing students perceive substance abuse as a complex issue influenced by diverse personal and environmental factors.	1.1 The multifaceted nature of substance abuse. 1.2 Personal factors that contribute to substance abuse. 1.3 Environmental factors that promote substance abuse.
2. Substance abuse impacts student nurses across three domains of their well-being.	2.1 Work effects 2.2 Academic effects 2.3 Psychological effects
3. The management of substance abuse within the education institution lacks comprehensive, structured and consistent strategies.	3.1 Predominance of punitive measures over supportive interventions for addressing substance abuse. 3.2 The necessity for the involvement of external stakeholders in ameliorating the ramifications of substance abuse among students.

**TABLE 4:** Pilot participant demographics.

Educator	Demographics	Student	Demographics
Educator 1	56-year-old with 19 years of experience	Student 1	2^nd^ year
Educator 2	38-year-old with 4 years of experience	Student 2	2^nd^ year repeat
Educator 3	61-year-old with 30 years of experience	Student 3	3^rd^ year
Educator 4	47-year-old with 8 years of experience	Student 4	4^th^ year
Educator 5	47-year-old with 12 years of experience	Student 5	4^th^ year
Educator 6	51-year-old with 12 years of experience	Student 6	4^th^ year

Note: Pilot focus groups on a remote main campus away from the primary study sites.

**TABLE 5:** Main participant demographics.

Particpants	Campus A	Campus B	Campus C
Educator 1	47-year-old with 9 years of experience	-	-
Educator 2	52-year-old with 12 years of experience	-	-
Educator 3	-	56-year-old with 19 years of experience	-
Educator 4	-	42-year-old with 4 years of experience	-
Educator 5	-	-	61-year-old with 25 years of experience
Educator 6	-	-	59-year-old with 20 years of experience
Student 1	2nd year	-	-
Student 2	2nd year	-	-
Student 3	2nd year	-	-
Student 4	4th year	-	-
Student 5	-	1st year	-
Student 6	-	1st year	-
Student 7	-	2nd year repeat	-
Student 8	-	3rd year	-
Student 9	-	3rd year repeat	-
Student 10	-	-	2nd year
Student 11	-	-	2nd year repeat
Student 12	-	-	3rd year
Student 13	-	-	3rd year repeat
Student 14	-	-	3rd year
Student 15	-	-	4th year

### Theme 1: Nurse educators and nursing students perceive substance abuse as a complex issue influenced by diverse personal and environmental factors

A common perception among nurse educators and nursing students across the three campuses was that substance abuse is a complex issue influenced by personal and environmental factors. The definitions and examples of frequently abused substances were discussed, along with their negative impacts on students’ personal and academic lives and on the nursing profession. Participants highlighted the difficulty of identifying substance abusers and remarked that substance abuse is often used as a coping mechanism.

#### Subtheme 1.1: The multifaceted nature of substance abuse

The complex nature of substance abuse is influenced by several factors, including the social backgrounds of nursing students facing substance misuse issues. The socio-demographic background of students plays a key role in patterns of substance abuse. Factors contributing to substance abuse include social issues such as unemployment, poverty, lack of familial support and academic pressure. Nurse educator participants emphasised the social and psychological challenges arising from students’ family backgrounds, which are linked to alcohol and substance abuse problems. A nurse educator stated:

‘You find out when you sit with some of these students that there are psychological problems from their home backgrounds. Some have personal issues that lead to them drinking alcohol and abusing drugs.’ (ELNE: 46–50)

‘There is no support at home from the parents.’ (FG2:P1: 59–62)

A nursing student participant said:

‘For instance, family problems … I’ve got so many emotional scars and psychological issues that I’ve been through on my own and that I’ve never really had the platform to talk about.’ (FG2:P2:47–49)

Substance abuse is often used as a maladaptive coping mechanism by students experiencing stressors in their academic and personal lives. Nursing student participants shared that substances are frequently relied upon to deal with emotional, social, academic or personal challenges. They expressed concerns about the heavy workload in both academic and clinical settings, further worsened by a lack of recreational options for students. This situation causes significant stress, leading to substance use during weekends and after work hours, particularly as they are young individuals. Quotations from two nursing student participants illustrate these points:

‘You know we go through a lot at home … we go through a lot of emotional trauma and problems, and I feel like substance is an avenue to find a sense of belonging, whereby you can be around people and just talk because there is no support from home.’ (FG3:P2s:40–43)

‘Most of us as students experience substance abuse due to personal problems or work problems – at school or at workplaces.’ (FG3:P3s:143144)

The lack of work–life balance significantly contributes to alcohol and substance abuse. All student participants interviewed across campuses identified the absence of work–life balance as a contributing factor to substance abuse. They expressed that the academic workload, both in lectures and clinical settings, was overwhelming. This is a vicious cycle as substance abuse causes further lack of coping and work–life balance.

#### Subtheme 1.2: The personal impact of substance abuse

Nursing students identified several personal impacts of substance abuse, including non-disclosure of the nature of the problem by users, fear of judgement and expulsion. Fear of consequences also contributes to non-disclosure. This study showed that students who misuse substances often avoid revealing their issues because they fear being judged by their peers and nurse educators. They are worried that disclosing might lead to disciplinary measures or even expulsion from the educational institution. The following verbatim quotations illustrate these perceptions:

‘It’s too stiff [*referring to rigid rules*] at the education institution really … If we could feel more open to go to our lecturers or management with our problems, then it would make it a lot easier …’ (FG1:P2:411–412)

‘There was a case last year of students who were caught by our lecturers having this problem, they were suspended, and they were given that stigma. So, it’s not easy for us even if we do experience these kinds of problems to voice out because we are fearing the judgement …’ (FG1:P1:2325)

Additionally, they felt that there were no recreational opportunities provided to help ease the pressure. Consequently, young students turn to using substances over the weekends or after work to cope:

‘What should we also take into consideration is that, when we use substances to cope or to balance out our student life and our leisure time. …’ (FG2:P2:215–221)

‘… [*T*]here is no recreation, there is no leisure time, there is no place where you feel one with different people. … And I think it goes onto the fact that we are stuck between being students and being workers at the same time, we don’t know how to uhm.’ (FG2: P4:230–236)

Students also reported feeling judged by nurse educators when suspected of substance abuse.

‘Yes, they [*educators*] just react: “Ok, if, you don’t come to class because of drugs, then go home.” … you are doing drugs, then you are going home” [*being expelled*]. It’s like that.’ (FG2s:33–35)

It confirms that nursing students face a range of challenges during their training.

Nurse educators observe students exploring independence and freedom. When students live away from home for the first time, they often explore their independence and engage in risky behaviours, including substance use:

‘From my perception, when students are living far away from their parental guidance and control, they start to explore independence and freedom from parental or adult supervision, some for the first time in their lives, they then start experimenting with many lifestyles, for example drinking alcohol and using substances.’ (FG2:P2s:127–128)

Students themselves described using substances to relax and cope with the stresses of the week:

‘On a Friday, I really forget about books and then go to wherever or get something to drink, just to relax and let go of whatever was going on this whole entire week. You don’t know whether you are coming or going and all that, and then you just need temporary happiness at that moment … If it’s weekends, we overdo it, if it’s holidays, we overdo it.’ (FG1:P2:127–128)

#### Subtheme 1.3: Environmental factors that promote substance abuse

Nurse educators indicated that environmental factors play a significant role in student substance abuse. These include poorly managed accommodation, proximity to taverns and liquor outlets, peer pressure and the availability of substances such as alcohol, cannabis, crystal methamphetamine and cocaine.

*Access to substances such as alcohol and drugs* is easily available because of the environment where nursing students live. Students reported being able to access substances easily at nearby liquor stores, taverns and taxi ranks. Educators observed that some accommodations are in high-risk areas, which are not conducive to learning. Nurse educators say:

‘This is a long-standing problem. It’s coming from social problems; unemployment, poverty and because they have nothing to do, so and because liquor is easily available.’ (PE1: 95–100)

Nursing students say:

‘Because it [*alcohol*] is free for us to use, you know it’s there. We can use it anytime and management will not even know.’ (FG2:P2s: 226–228)

Alcohol and drugs were exchanged in dorm rooms and classrooms, either to escape academic stress or to boost performance. *Inability to cope with clinical exposure* is an overwhelming stressor that worsens substance abuse. The researcher discovered that students found it difficult to manage the pressures and traumatic experiences they faced during clinical placements, particularly in surgical wards. The abrupt shift from lockdown to clinical demands, including making up missed work and tests, increased their stress. This quote is from a student nurse:

‘… [*T*]hings that we see, we sometimes find it hard to cope … So, exposure to clinical areas also contributes to our inability to cope and turn to substance abuse. I also think that the pressure after lockdown such as writing tests, remedials and doing skills … to relieve those stresses, they use alcohol or drugs.’ (FG3: P4s: 38–47)

The combination of internal and external demands generates pressure for students to constantly perform at their best, leading to emotional and mental distress. *Poorly managed accommodation* makes it easy for students to access substances. The lack of supervision in student housing was also identified as a significant factor in substance abuse. Nurse educators expressed concern about students residing in areas close to taverns and other risky locations:

‘They [*the students*] are scattered in the locations, around the taverns, in the stations, and at the other places that are not conducive to their learning. There it makes matters worse …’ (PNE2:201–205)

‘It came up to our knowledge, that they [*the students*] are bringing alcohol to the nurses’ home….’ (ELNE2:131–133)

These concerns highlight the urgent need for safe, structured student accommodation to help curb substance abuse and create a supportive learning environment.

### Theme 2: Substance abuse impacts student nurses across three domains of their well-being

When nurse educators and student nurses highlighted the impact of substance abuse on the well-being of nursing students, three subthemes arose. The first concerned the physical effects of substance abuse, the second the social effects and the third focused on the psychological effects on students’ studies, work and behaviour. These subthemes are discussed below.

#### Subtheme 2.1: Work effects

Nurse educators expressed concern about the physical appearance of students affected by substance abuse. They described these students as appearing untidy, dirty or having poor personal hygiene, with some even smelling of alcohol. Certain students admitted experiencing weight loss because of substances like *Tik* (street name for crystal methamphetamine). This is evident in the following quotations. A nurse educator recalls:

‘A few students are coming very late, not doing their work very well, and even are scruffy looking….’ (PE1:21–23)

A student nurse explained:

‘I lost so much weight, you know, because of Tik [*street name for crystal methamphetamine*]. It makes you lose weight.’ (FG2:P21:21–23)

#### Subtheme 2.2: Academic effects

Substance abuse also adversely affected academic engagement and performance. Nurse educators observed high absenteeism across both classes and clinical placements, resulting in poor academic performance and test failures. Students arrived late, slept in class or disrupted others, prompting referrals to management. Although some students were referred to employee assistance programmes, disciplinary actions ensued when they failed to meet academic requirements, such as the 85% attendance threshold. Some were expelled after repeated violations and unsuccessful attempts at rehabilitation, including psychiatric referrals. Nurse educators elaborated on these issues:

‘Substance abuse is a real problem in the education institution. It makes us so concerned, so worried about our students. It causes absenteeism … When we were observing or assessing the students, it interferes with their mindset, and thought processes … it interferes and our fear, my fear, my main fear, the student does not focus. They lose the ability to focus, the ability to grasp the knowledge, and they lose concentration, …’ (ELNE4:22–29)

‘It is impacting negatively on their performance academically even if the student was a performing student. Because of drug abuse you find that the performance is dropping. It’s sad that some of them drop out.’ (ELNE1: 51–54)

The impact of substance abuse on professionalism raised concerns about adherence to the nursing ethical code. Nurse educators were alarmed by the misconduct of affected students, which brought discredit to the educational institution. Incidents included disrespectful behaviour, fights among students and violations of nurses’ home policies, such as bringing alcohol or drugs onto the premises. These events led to police involvement, and the nurses’ home eventually became uninhabitable and was closed. The following quotations illustrate this:

‘There is a big case of, what was last year? We were doing first year, of students that went for a night out, they came back to the nurses’ home, they stabbed each other, and the other one ended up being in the ICU.’ (PE3s: 85–87)

#### Subtheme 2.3: Psychological effects

Nurse educators also observed what they interpreted as psychological effects of substance abuse, including erratic behaviours such as crying, hallucinations and physical altercations. These behaviours disrupted the classroom and clinical environments. Nurse educators shared:

‘You find that whoever is using a substance abuse, more especially if you must concentrate on the students, they tend to be disrespectful.’ (PE1:44–46)

Another nurse educator said:

‘… [*T*]hat tends to modify people’s perceptions or the people’s brain and display another personality … of which I think it modifies someone’s perception or someone’s character if I can put it that way.’ (PE2s:7–11)

Psychological effects are complex factors influencing alcohol and drug abuse behaviours, including individual susceptibility to alcohol and other substances.

### Theme 3: The management of substance abuse within the education institution lacks comprehensive, structured and consistent strategies

Nurse educators and nursing students highlighted the inconsistent, uncoordinated and incomplete support and interventions provided to students dealing with substance abuse issues by educational institution administrations. While some nurse educators took it upon themselves to counsel affected students, this counselling was sporadic and lacked a structured approach or guiding framework. Nursing student participants expressed their dissatisfaction with the absence of dedicated facilities on campus for managing substance abuse, feeling that the response was more about punishment than offering support. This topic further developed into two subthemes, which are explored in the subsequent discussion.

#### Subtheme 3.1: Predominance of punitive measures over supportive interventions for addressing substance abuse

Both nurse educators and nursing students highlighted the lack of supportive measures to address substance abuse, noting a reliance instead on punitive actions for those affected. The following paragraph includes direct quotes from nursing students that emphasise these observations. Nursing student participants commented:

‘There is no help other than punishment for this. … If someone is referred to a hospital or psychiatric institution, it will be the family, not the education institution. … If you have misbehaved due to substance abuse in the education institution, you will be dismissed or face some sort of punishment.’ (FG3s: P1 and P3 111–114; 128–130)

A nurse educator participant replied:

‘There are no strategies or any way of identifying those students and managing them before they use drugs, and even when we find that they are using them … There is no on-site social worker or psychologist, and there is no proper referral system….’ (ELNE1: 109–113)

#### Subtheme 3.2: The necessity for the involvement of external stakeholders in ameliorating the ramifications of substance abuse among students

During the focus group interviews, both nurse educators and nursing students voiced concerns about the lack of external professional support, such as an on-site student support centre staffed by psychologists and social workers, to address substance abuse challenges within the educational institution. Participants from both focus groups highlighted that some students arrive at the institution already facing issues rooted at home, issues that are often more complex than they initially appear and require intervention from trained professionals for counselling and therapy. A notable challenge was the role of nurse educators acting as student counsellors, which led to inconsistent supervision and follow-up. Direct quotes from a nurse educator participant reinforce these points:

‘We as lecturers have been advocating for a psychologist because when you sit down with some of these students, you find that there is a psychological problem emanating from home.’ (ELNE1:46–47)

‘If we could have a designated on-site student support centre … that could be of help. I am talking about a student support centre that is going to be staffed by psychologists and social workers so that students know exactly where to go when problems arise. We really need stakeholders who can be available daily to assist our lecturers with these types of problems.’ (ELNE2: 206–207; 210–212)

A nursing student deliberated:

‘I think we should be provided with counsellors, people who are trained, because most of us experience substance abuse because of personal problems or work problems. So, we would benefit from counselling sessions every now and then; this could help prevent substance abuse….’ (FG3s:142–146)

## Discussion

The findings of this study align with existing literature, which emphasises that the stress experienced by students in educational institutions often manifests as psychological distress, potentially leading to alcohol and drug misuse (Barbayannis et al. 2022). Substance abuse among students is a multifaceted issue shaped by a complex interplay of personal and environmental factors. Many students grapple with psychological challenges stemming from their home environments, which often act as catalysts for substance use (Lewis et al. 2021). For some, alcohol and drugs become maladaptive coping mechanisms for managing overwhelming stressors, including academic pressure and clinical exposure, particularly in health-related fields (Brito et al. 2024; Hirani et al. 2022). Veesart and Cannon (2022) report that drinking leads to missed classes, poor grades, injuries and cognitive impairment. Volkow and Blanco (2023) similarly found that alcohol, cannabis and cocaine use resulted in aggression, depression, anxiety, paranoia and cognitive decline. The educational environment itself frequently lacks comprehensive, structured strategies to address substance abuse. Instead, this public nursing institution reverts to punitive measures in the absence of a structured support system for students, which can deter students from seeking help because of fears of disciplinary action or expulsion (El Hayek et al. 2024). This punitive approach fails to provide the support needed to tackle the root causes of substance use, such as mental health struggles and external pressures (Kong et al. 2023). Work–life imbalance is a critical factor in substance abuse. Students often face relentless academic demands while managing personal and social responsibilities, leading to emotional and mental distress (Jiang et al. 2022). The combination of internal expectations and external demands creates significant pressure, which some students seek to relieve through substance use. Drinking the day before work can lead to either a decline in clinical performance, posing risks to patient safety and quality of care (Zolkefli 2024) or absence from work the next day (Duckworth et al. 2023). This observation underscores the urgent need for early intervention and ongoing support within nursing education programmes (Zolkefli 2024). This not only affects their physical appearance, with nurses appearing untidy, but also leads to erratic behaviour and impaired academic engagement and performance (Brito et al. 2024; Duckworth et al. 2023).

Environmental factors, including housing instability and stigma associated with substance use disorders, further exacerbate the problem (El Hayek et al. 2024; Lewis et al. 2021). This stigma creates barriers to disclosure and to accessing support services, reinforcing a cycle of isolation and abuse. Nurse educators, who often serve as informal counsellors, struggle to provide consistent supervision and follow-up because of these systemic gaps (Hirani et al. 2022). Overall, there is an urgent need for the public nursing educational institution to adopt comprehensive, consistent strategies that integrate mental health support, reduce stigma and promote healthy coping mechanisms. Developing evidence-based prevention models tailored to nursing students could strengthen institutional efforts to reduce substance abuse (Netshiswinzhe et al. 2023). These measures should replace punitive responses and foster a supportive environment in which students can seek help without fear, ultimately improving both their well-being and academic success (El Hayek et al. 2024; Kong et al. 2023).

### Strengths and limitations of the study

The richness of the data provided insights into the perceptions of nurse educators and student nurses regarding substance abuse and its management at the nursing education institution under study, enabling the researcher to achieve the research goals. The researcher spent sufficient time in nursing education to understand the context in which the research study was conducted. A limitation of the study is that sub-campuses, private nursing education institutions and universities were not included. As a result, the findings cannot be generalised. A broader range of views might have been elicited if the sample had included sub-campuses. Although confidentiality was maintained during the interviews, participants’ anonymity was challenging because the focus group discussions were conducted during block time. Staff who were not involved in the study were aware of the participation of nurse educators and student nurses in the research, which could have affected the participants’ ability to share openly and freely.

### Recommendations

Recommendations to address substance abuse among student nurses at the nursing education institution emanated from participants and evidence-based literature findings. These are summarised according to the themes identified in the research:

### Recommendations from theme 1 and theme 2

A dedicated centre, staffed by psychologists and social workers, should be established to provide confidential counselling and support for both students and staff. Self-awareness campaigns should include regular talks and workshops, incorporated into student orientation and offered throughout the academic year to educate students on the risks of substance use and effective coping strategies. Pre-admission and periodic screening for substance use should be introduced at entry and at regular intervals to enable early identification and support for affected students. Standardised procedures for managing substance abuse cases should be developed to ensure consistency and fairness in response. Additionally, student accommodation should be improved with the provision of safe, well-monitored housing to reduce exposure to environments that may encourage substance use.

### Recommendations from theme 3

Structured intervention strategies are necessary, including the development of systematic, institution-wide programmes to tackle substance abuse, benchmarked against best practices from other institutions. A shift from punitive to supportive measures is crucial, with emphasis on rehabilitation and student retention. Remedial support should be provided before disciplinary actions are taken. Additionally, a formal substance abuse protocol should be established, outlining clear, step-by-step procedures for managing such cases.

### Recommendations for future research

Future studies should be extended to include sub-campuses and other nursing institutions. In addition, including education institution staff as participants would provide a broader understanding of the dynamics of substance abuse within the nursing education environment. Collaborative efforts are recommended to develop and assess protocols for substance abuse management in nursing education institutions.

## Conclusion

The absence of standardised protocols has led to inconsistent, reactive case management, which compromises student retention. The nursing college is inadequately equipped to deal with substance use among students, and a comprehensive, structured and proactive institutional framework is urgently needed. Substance use among nursing students is fundamentally a health and well-being issue that requires professional and structured interventions. The insistence that support should come before disciplinary action concludes that the current sequence is reversed, with consequences being imposed before help is even offered. The clear call for a shift from punitive to supportive measures directly suggests that disciplinary-first approaches have not effectively addressed substance abuse and may, in fact, be making outcomes worse by pushing affected students out of the system rather than helping them recover. A holistic, proactive approach encompassing early screening and rehabilitation-focused support is essential to protect students’ academic success and well-being and to prevent avoidable attrition from the nursing profession.
